# Prognostic utility of the acute cardiac ischemia time-insensitive predictive instrument (ACI-TIPI)

**DOI:** 10.1186/1865-1380-4-49

**Published:** 2011-07-31

**Authors:** Jonathan S Ilgen, Alex F Manini, Udo Hoffmann, Vicki E Noble, Ediza Giraldez, Supapan Nualpring, J Stephen Bohan

**Affiliations:** 1 Division of Emergency Medicine, University of Washington School of Medicine, Harborview Medical Centre, 325 9th Avenue, Box 325709, Seattle, WA 98104, USA; 2 Department of Emergency Medicine, Mt. Sinai School of Medicine, 1 Gustave L. Levy Place, Box 1620, New York, NY, 10029, USA; 3 Cardiac MR PET CT Program, Massachusetts General Hospital, Harvard Medical School, 45 Fruit Street, Boston, MA, 02114, USA; 4 Division of Emergency Medicine, Massachusetts General Hospital, Harvard Medical School, 45 Fruit Street Boston, MA, 02115, USA; 5 Department of Emergency Medicine, Brigham and Women's Hospital, Harvard Medical School, 75 Francis Street, Boston, MA, 02115, USA

## Abstract

**Background:**

We sought to evaluate the test characteristics of the acute cardiac ischemia time-insensitive predictive instrument (ACI-TIPI) in relation to 30-day major adverse cardiac events (MACE) among patients who presented to the Emergency Department with symptoms suggestive of an acute coronary syndrome. We then examined the test characteristics of various dichotomous ACI-TIPI cut points.

**Methods:**

We prospectively recruited a cohort of Emergency Department (ED) patients with acute chest pain at two urban university hospitals between June and September 2006. Upon enrollment, baseline demographics and cardiac risk factors were collected. An electrocardiogram (ECG) was performed and analyzed with the built-in ACI-TIPI multiple regression model software. An ACI-TIPI probability score was recorded for each patient. Diagnostic test characteristics of ACI-TIPI for MACE (non-ST elevation myocardial infarction (NSTEMI), percutaneous coronary intervention, coronary artery bypass grafting, and all-cause mortality) within 30 days were determined.

**Results:**

Of 144 patients enrolled (mean age 59.1 ± 14.1 years, 59% men), 19 (13%) patients suffered MACE within 30 days. Receiver-operating characteristics (ROC) for ACI-TIPI yielded a c-statistic of 0.69 (95% CI 0.59-0.80, *p *< 0.01). An ACI-TIPI score of ≥ 20 had 100% sensitivity (95% CI 82-100), 100% negative predictive value (95% CI 86-100), and 21% specificity (14-31%).

**Conclusions:**

These preliminary results suggest that, while ACI-TIPI has limited discriminatory value for MACE overall, a score of < 20 may have 30-day prognostic utility to allow for safe outpatient management in patients with acute chest pain.

## Background

Over 6 million patients undergo evaluation for chest pain in the United States each year [1,2]. A large subset of these patients will have a diagnosis other than an acute coronary syndrome (ACS), while 1-5% of these patients will be inappropriately discharged with true myocardial infarctions [3,4]. Misinterpretation of electrocardiogram findings has been cited as a major contributor to missed myocardial infarctions in the emergency department (ED) [3]. Patients who are inappropriately discharged have a mortality rate that is nearly twice that of patients who are admitted [4]. Unfortunately, efforts to identify these high-risk patients by historical data and available risk scores have proven unreliable [5-7]. To maintain both safety and efficiency, recent work had addressed patients at very low risk for ACS who may not require further testing [8], highly sensitive cardiac biomarkers [9-15], rapid imaging modalities for cardiac risk stratification [16-20], and post-risk stratification scores that incorporate demographic and clinical data [21]. In this context, a highly sensitive, rapid, and non-invasive risk assessment tool would improve the triage of patients with chest pain, especially in low-risk ED populations. Furthermore, such a tool could prove to be useful in settings where biomarkers and advanced technology are not readily accessible.

The acute cardiac ischemia time-insensitive predictive instrument (ACI-TIPI) was developed as a means to risk-stratify patients in real time [22,23]. Subsequent studies have demonstrated that this tool accurately estimates the risk of acute cardiac ischemia in an undifferentiated patient population, and substantially speeds decision making and triage by novice clinicians [22,24-26]. When used in a low-risk population of patients with chest pain admitted to an observation unit, a dichotomous ACI-TIPI cut point of 20% or greater was shown to be predictive of nonnegative exercise treadmill tests [27]. Two systematic reviews of available cardiac risk stratification technologies by the National Heart Attack Alert Program have graded the evidence supporting ACI-TIPI as Class A, one of only three tools to receive this rating [28].

The ability of an ACI-TIPI cut point to identify patients at very low risk of myocardial infarction (less than 2% at 45 days) has been studied in patients admitted to the hospital or chest pain observation units [8,22,25,29], but not as a prognostic indicator in an undifferentiated cohort presenting with symptoms concerning for myocardial infarction. Identification of patients with chest pain who are at low-risk for cardiovascular events at 30 days may improve decision-making regarding initial triage and disposition. The primary objective in this prospective multicenter study is to define an optimal ACI-TIPI cut point that can predict 30-day major adverse cardiac events (MACE) in patients with acute chest pain.

## Methods

### Study setting and design

A prospective cohort study of patients with acute chest pain was conducted at two urban university emergency departments, each with over 55,000 visits per year. Both institutions are staffed 24 hours a day with board-certified emergency doctors. The study protocol was approved by each hospital's institutional review board, and all patients provided written informed consent.

### Study population

We enrolled consecutive ED patients with symptoms consistent with myocardial ischemia during periods of research assistant availability, on weekdays during business hours (9 a.m. to 5 p.m.), between June and September 2006. All patients were evaluated by a board-certified emergency physician and received standard treatment irrespective of their participation in the study. We included all patients with chest pain or symptoms consistent with myocardial ischemia (e.g., shortness of breath). All patients had a single troponin measurement as part of their workup. Because this was an observational study, there was variability among treating physicians regarding the decision to measure serial biomarkers and obtain functional cardiac studies. We excluded patients with obvious alternative diagnoses who did not receive any workup for myocardial ischemia, as well as patients with ST elevations on their initial electrocardiogram, as we did not want the research protocol to interrupt expeditious transfer to the catheterization suite. Assuming a baseline 20% prevalence of the predictor (ACI-TIPI cut point) and a 5% MACE rate, we calculated the need to include 156 patients to have 80% power and 5% alpha to show a two-fold risk difference.

### Study protocol

Patients meeting the enrollment criteria above were approached by trained research assistants. After providing written informed consent, a standardized data collection instrument was completed by the research assistant. This instrument assessed baseline demographics, traditional cardiac risk factors (diabetes, hypertension, hyperlipidemia, smoking history, and history of coronary artery disease in a first-degree relative), and current medication use. These data were recorded and entered into the study database.

Upon enrollment, patients had an ECG performed, which was analyzed with the built-in ACI-TIPI multiple regression model software. To do this, a research assistant acquired and entered the following data required for the ACI-TIPI program: the patient's age and sex, the presence of chest or left arm pain, and whether these symptoms were their primary complaint. Based on ECG evidence of pathologic Q waves and ST- and T-wave changes, the software calculated a probability score for myocardial infarction, reported as a percentage, which represents the probability of acute ischemia. Exact numerical values for these log-odds ratios are reported elsewhere [30].

The diagnosis of myocardial infarction was based upon European Society of Cardiology/American College of Cardiology (ESC/ACC) guidelines as a typical rise and fall of troponin T (Elecsys 2010, Roche Diagnostics, Indianapolis, IN) or troponin I (Advia Centaur, Bayer Healthcare, Tarrytown, NY) in the setting of ischemic symptoms or characteristic ECG changes such as pathologic Q waves or ST depression [31].

### Outcomes

The primary outcome was any MACE within 30 days of evaluation, including these events during the index visit. MACE was defined as any one of the following: non-ST elevation myocardial infarction (NSTEMI) as defined by ESC/ACC criteria [31], percutaneous coronary intervention, coronary artery bypass grafting, and all-cause mortality. Non-ST elevation myocardial infarction was defined as the ''detection of rise and/or fall of cardiac troponin with at least one value above the 99th percentile of the upper reference limit together with evidence of myocardial ischemia with at least one of the following: (1) symptoms of ischemia, (2) ECG changes indicative of new ischemia (new ST T changes or new left bundle-branch block), (3) development of pathological Q waves in the ECG, and (4) imaging evidence of new loss of viable myocardium or new regional wall motion abnormality" [31]. These events were recorded by review of subsequent hospital records, scripted follow-up phone calls, and searches of the Social Security Death Index. The secondary outcome of in-hospital non-ST elevation myocardial infarction was agreed upon by consensus of two study investigators to better ensure the validity of this measurement.

### Statistical analysis

We calculated sensitivities, odds ratios (ORs), and 95% confidence intervals. Descriptive statistics were used for baseline characteristics. χ^2 ^and Student's *t *tests were used to compare categorical and continuous variables, respectively. All *p*-values were two-tailed with a value less than 0.05 considered significant. Area under the receiver-operating characteristics curves (AUCs) of ACI-TIPI to predict MACE were plotted, to assess overall discrimination and to define sensitivity and specificity for various cut points of the ACI-TIPI score (e.g., 10, 20, 30, and 40). A dichotomous cut point was chosen as the ACI-TIPI score that achieved a sensitivity and negative predictive value (NPV) of 100%. Data were analyzed using SPSS version 14.0 (SPSS, Inc., Chicago, IL).

## Results

We enrolled 144 patients who met the inclusion criteria. Eight patients (5.5%) were lost to follow-up at 30 days, yielding 136 patients with complete data. The clinical characteristics of patients with and without MACE are summarized in Table 1. Overall, there was a 30-day MACE rate of 13.8% (19 patients), with 2 deaths (1.4%). Seventeen of the patients with MACE underwent coronary angiography with subsequent stent placement or coronary artery bypass grafts. Of the two remaining patients, one received medical management, and one expired from indeterminate causes.

**Table 1:** Baseline clinical characteristics

Parameters	No. or mean (% or SD)	MACE (% or SD)	No. events (% or SD)
Mean age	59.1 (± 14.1)	68.8	57.7
Male sex	85 (59.0)	12 (62.2)	69 (58.8)
Age > 55	85 (59.0)	17 (89.4)	28 (23.9)
Cardiac risk factors			
Smoking	20 (13.0)	1 (5.3)	18 (15.4)
Hypertension	75 (52.1)	12 (63.2)	58 (49.6)
Hypercholesterolemia	64 (44.4)	11 (57.9)	49 (41.9)
FH	42 (29.2)	3 (15.8)	38 (32.5)
DM	23 (16.0)	2 (10.5)	20 (17.1)
NSTEMI	10 (6.9)	10 (52.6)	0
30-day MACE*	19 (13.2)	NA	NA
Total	144	19	117

MACE, major adverse cardiac events [non-ST elevation myocardial infarction (NSTEMI), percutaneous coronary intervention, coronary artery bypass grafting, and all-cause mortality]; NA, not applicable; SD, standard deviation; FH, family history of coronary artery disease; DM, diabetes mellitus; NSTEMI, non-ST elevation myocardial infarction. *Eight patients were lost to follow-up.

**Figure 1:**
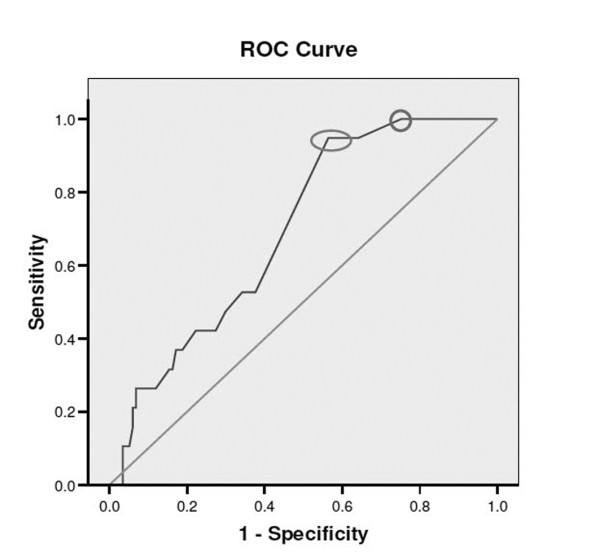
**Receiver-operating characteristics (ROC) of ACI-TIPI for prediction of MACE**. The *black curve *represents the ROC of ACI-TIPI for prediction of 30-day major adverse cardiac events (MACE), defined as non-ST elevation myocardial infarction (NSTEMI), percutaneous coronary intervention, coronary artery bypass grafting, and all-cause mortality. The *gray line *is the reference line. The *circle *and *oval *represent ACI-TIPI cut points of 20% and 30%, respectively.

An ACI-TIPI score above 20% was associated with a 20% increase in the odds of having a MACE within 30 days, while an ACI-TIPI score above 30% was associated with a 13 times greater odds of having a MACE (Table 2). The discriminatory power of the ACI-TIPI yielded an AUC of 0.69, as shown in Figure 1. The test characteristics for ACI-TIPI at various cut points are shown in Table 3. A cut point of 20 percent achieved a sensitivity and negative predictive value of 100 percent for MACE with a specificity of 21%. In contrast, with a cut point of 30 percent, the specificity increased two-fold to 40%, while maintaining a sensitivity of 94% and a negative predictive value of 98%.

**Table 2:** ACI-TIPI and 30-day MACE

		**MACE**			**χ^2^**	**OR (95% CI)**
		**No**	**Yes**	**Total**		
	
**ACI-TIPI (%)**	< 20	25	0	25	*P *= 0.024	1.2 (1.1-1.3)
	≥ 20	92	19	111		
	< 30	51	1	52	*P *= 0.001	13.9 (1.8-107.7)
	≥ 30	66	18	84		

ACI-TIPI, Acute cardiac ischemia-time insensitive predictive instrument; MACE, major adverse cardiac events [non-ST elevation myocardial infarction (NSTEMI), percutaneous coronary intervention, coronary artery bypass grafting, and all-cause mortality]; OR, odds ratio; CI, confidence intervals

**Table 3:** Test characteristics of ACI-TIPI to predict 30-day MACE

ACI-TIPI Score (%)	**No**.	Sensitivity (95% CI)	Specificity (95% CI)	PPV (95% CI)	NPV (95% CI)	LR+	LR-
≥ 10	120	100 (82-100)	13.7 (8-21)	15.8 (10-24)	100 (79-100)	1.2	0
≥ 20	111	100 (82-100)	21.4 (14-30)	17.1 (11-25)	100 (86-100)	1.3	0
≥ 30	84	94.7 (74-100)	43.6 (34-53)	21.4 (13-32)	98.1 (90-100)	1.7	0.1
≥ 40	39	42.1 (20-67)	73.5 (65-81)	20.5 (9-36)	88.7 (81-94)	1.6	0.8

ACI-TIPI, acute cardiac ischemia-time insensitive predictive instrument; MACE, major adverse cardiac events (non-ST elevation myocardial infarction (NSTEMI), percutaneous coronary intervention, coronary artery bypass grafting, and all-cause mortality); CI, confidence intervals; PPV, positive predictive value; NPV, negative predictive value. LR+, positive likelihood ratio; LR-, negative likelihood ratio

## Discussion

In this preliminary study of ED patients with chest pain, ACI-TIPI from the presentation ECG had prognostic utility for prediction of 30-day MACE. ACI-TIPI scores greater than 20 percent were highly predictive of MACE at 30 days, with sensitivity and negative predictive values of 100 percent. If validated in larger studies, use of an ACI-TIPI cut point may define a low-risk subset of patients with acute chest pain amenable for outpatient management.

These data add to the current literature. In their 1998 multicenter study, Selker et al. evaluated the triage decisions made by physicians in the setting of electrocardiograms printed either with or without headings that denoted the calculated probability of ischemia [22]. Use of this technology decreased triage to higher acuity inpatient settings and increased the percentage of patients discharged home. Thirty-day mortality rates between intervention and control groups were similar. Our study differs from this work in two important ways. First, rather than supplementing physician judgment with calculated probabilities between 0 and 100 percent, a dichotomous cut point simplifies decision-making for the clinician. Second, this cut point aims to identify a cohort of patients with a lower 30-day adverse event rate--as opposed to prior studies that looked at mortality--in real time. Measuring major adverse cardiac events is more useful to emergency physicians, since overall mortality from ACS is so low.

Our results also differ from the initial prospective validation of ACI-TIPI by Selker et al. [23], which demonstrated a 5.3% rate of acute myocardial infarction within 48 hours among patients with ACI-TIPI scores between 0 and 25%. When measuring 48-h outcomes in low- to moderate-risk cohorts defined by ACI-TIPI scores between 0 and 25%, 5.3% of patients had an acute myocardial infarction. Though this study enrolled both admitted and discharged patients with chest pain, this differs from our study in that both cohorts had follow-up ECG and cardiac enzyme testing (CK-MB) at 48 hours, potentially explaining the difference in event rates when compared to our data. Similarly, Seyal et al. examined the probability of acute myocardial infarction across a range of ACI-TIPI scores, and found that values greater than 20% had a 97% sensitivity for this outcome, while a sensitivity of 98% could be achieved if ACI-TIPI values greater than 10% were used [29]. This study, however, only enrolled patients who were admitted to the hospital. This likely explains the higher event rate and lower sensitivity of ACI-TIPI seen in the data presented here. Thirty-day MACE was not assessed in either of these studies.

The aim of identifying a low-risk cohort of patients *a priori *is similar to the work of Mitchell et al. [8], though our patients were drawn from the general ED population rather than a preselected group that was enrolled upon transfer to a chest pain observation unit. Furthermore, our results are consistent with past data demonstrating that ACI-TIPI scores less than 20 are predictive of negative exercise stress tests, further suggesting that these patients are at lower risk for adverse outcomes [27].

The ACI-TIPI instrument was designed as a screening tool. As such, the low specificities demonstrated for each of the ACI-TIPI cut points in this study are acceptable. Additional data from the history and physical examination, cardiac biomarkers, and provocative testing can be used to better refine the specificity of these patients' workup. Table 3 also illustrates that both the PPV and LR+ for ACI-TIPI scores greater than 40 were lower than those with scores greater than 30. This is likely due to an inaccurate point estimate due to a smaller number of subjects with scores greater than this value, though these results would suggest that a cut point greater than 40 is less useful for the purpose of screening for 30-day MACE.

### Limitations

Interpretation of these results should incorporate several limitations. First, while an ACI-TIPI cut point of 20% achieved a sensitivity and negative predictive value of 100% for MACE, our study sample was relatively small, and the wide confidence intervals for each of these values preclude widespread application of this tool for safe triage decision-making. To obtain a 95% CI width of 10% around our point estimates (OR), given our MACE rate of 13%, we would have needed 43 MACEs. To accomplish these this number of events would require approximately twice the number of patients enrolled in this study. A larger study is needed to validate these findings. Second, enrollment of patients only during the hours of research assistant availability may have introduced an inclusion bias into our sample. Due to the broad constellation of complaints that could be interpreted as possible ACS, we were unable to retrospectively identify the overall number of patients who met our enrollment criteria and were not enrolled. However, the baseline characteristics of our study population reveal a quite heterogeneous sample with respect to cardiac risk factors. Our MACE rate was 13.8%, which is similar to prior studies of undifferentiated chest pain cohorts [9-11]. Third, because we have derived this optimal cut point from our data, we may have overestimated the diagnostic performance. Lastly, because this study was conducted at two large tertiary hospitals, caution must be taken before generalizing these results to other non-urban populations or community hospitals.

## Conclusions

These preliminary results suggest that ACI-TIPI is useful for 30-day prediction of MACE. When used in the setting of ED patients with acute chest pain, a dichotomous cut point of less than 20% may aid identification of patients amenable for outpatient management. A prospective cohort study with a larger sample size is warranted to confirm these findings.

## Competing interests

The authors declare that they have no competing interests.

## Authors' contributions

JI participated in the design of the study, provided oversight for the data collection, performed the 30-day follow-up data collection, and drafted the manuscript. AM participated in the design of the study, provided oversight of the 30-day follow-up data, performed the statistical analyses, and edited the manuscript. UH participated in the study design and edited the manuscript. VN participated in the design of the study, provided oversight of the primary data collection, and edited the manuscript. EG and SN performed the primary data collection. JSB conceived the original study design and edited the manuscript. All authors read and approved the final manuscript.
